# Factors associated with father–infant bonding during the COVID-19 pandemic: an internet-based cross-sectional study in Japan

**DOI:** 10.1038/s41598-023-40225-2

**Published:** 2023-08-22

**Authors:** Etsuko Nishimura, Rina Shoki, Mika Kato, Daisuke Yoneoka, Sumiyo Okawa, Takahiro Tabuchi, Erika Ota

**Affiliations:** 1 Global Health Nursing, Graduate School of Nursing Science, St. Luke’s International University, 10-1 Akashi-cho, Chuo-ku, Tokyo, 104-0044 Japan; 2 Center for Surveillance, Immunization, and Epidemiologic Research, National Institute of Infectious Diseases, Tokyo, Japan; 3 Institute for Global Health Policy Research, Bureau of International Health Cooperation, National Center for Global Health and Medicine, Tokyo, Japan; 4 Cancer Control Center, Osaka International Cancer Institute, Osaka, Japan; 5 Tokyo Foundation for Policy Research, Tokyo, Japan

**Keywords:** Epidemiology, Risk factors

## Abstract

The COVID-19 pandemic has forced lifestyles changes and affected the relationships between fathers and their infants. However, the factors associated with paternal–infant bonding have not been clarified. This study aimed to explore the factors associated with father–infant bonding during the COVID-19 pandemic in Japan. This cross-sectional study used data from a nationwide survey and the Japanese version of the Mother-to-Infant Bonding Scale (MIBS) to measure father–infant bonding. The participants were divided into two groups depending on their partners’ parity. A linear regression model (Gauss–Markov-type) was used for both groups. A total of 1055 men were included in the analysis. Of these men, 521 (49.4%) had a primipara partner, and 534 (50.6%) had a multipara partner. No significant differences were found between the two groups’ MIBS-J scores. Fathers’ mental health, relationship with the partner and family members, abusive behavior towards children, wanted pregnancy, and the youngest child’s Neonatal Intensive Care Unit admission history were associated with father–infant bonding. Regarding factors related to COVID-19, caring for the child while the partner is at home has a negative impact on bonding, while fear related to infection with COVID-19 has no negative impact on bonding.

## Introduction

Bonding is defined as the development of a core relationship between parents and infants; the process after birth has a tremendous impact on the future development of the child^1–3^. Fathers play an important role in the psychological development of mothers and children and contribute to a child’s genetic substrate of psychological health^4^. It has been suggested that fathers, as well as mothers, should play an active role throughout pregnancy and childbirth, promoting father–infant bonding and increasing fathers’ parenting time^5^. Furthermore, both mothers and fathers undergo mental changes during the gestation period^5^. A study conducted in Japan showed that approximately 10% of the fathers had postpartum depression^6^. Mental health issues such as paternal stress and anxiety are related to bonding between fathers and their children^7–10^. In addition, attendance at birth^11,12^, parental leave^13^, workload^10^, and marital relationships^8,9,14^ have been reported as factors related to father–infant bonding in previous studies. Furthermore, a study examining factors related to mother–infant bonding and father–infant bonding also found that the fact that a child is the first child for the mother also affects the bond between that child and the father^8^.

The COVID-19 pandemic has forced lifestyle changes and affected the relationships between father and infant^15^. In Japan, a state of emergency was first declared in April 2020, and three emergency measures were implemented^16^. The current study was conducted while COVID-19 was becoming prevalent, and hospital capacity and healthcare infrastructure remained strained. During this period, a third state of emergency was declared in Japan^16,17^. The Japanese government had been encouraging companies to introduce telecommuting as a countermeasure against the new coronavirus^18^, leading to situations in which both parents work at home. This COVID-19 pandemic situation in Japan has led to an increase in social isolation, defined as less frequent contact with people other than co-residing family members, which has been greater among men than among women^19^. Shigemura et al.^20^ have also reported that the COVID-19 pandemic and the subsequent time at home has affected mental health.

As for children, the government requested that nursery schools stay open, in principle, even when the third state of emergency has been declared. However, if children are to be in close contact with a child infected with COVID-19, their parents are requested by the municipality to refrain from allowing them to attend nursery school^21^. Therefore, there may have been more occasions than usual for fathers to look after their children at home, even when their wives or partners were also at home. Additionally, staying home increases children’s emotional problems, which may cause parenting stress in fathers and worsen their relationships with their children^15^.

These situations could lead to serious problems such as domestic violence and abuse^20^. Although COVID-19 affects mother–child bonding^22^, the impact of stress on the father-child relationship is reported to be greater than that on the mother–child relationship^23^. Thus, the COVID-19 pandemic may have negatively impacted the bonding between fathers and their children.

During the COVID-19 pandemic, partner attendance at infant deliveries was not recommended so as to aid infection prevention in Japan^24^. Fathers play an important role during childbirth, and childbirth experience has a great impact on the subsequent childrearing of fathers^25^. Furthermore, a positive birth experience has a profound effect on subsequent childbearing^26^. Drastic restrictions on partner attendance have caused additional anxiety in mothers, which may have hindered early attachment between fathers and their children^27,28^.

Identifying factors associated with father–infant bonding is important because bonding problems can cause dysfunctional parenting, such as child abuse and neglect^2^. Our hypothesis is that the COVID-19 pandemic may have negatively affected the bond between fathers and their children. In addition to changes in the social environment, such as working patterns and economic conditions, father–infant bonding may continue to affect the father-child relationship even after the COVID-19 pandemic. However, the factors associated with the father infant-bonding in Japan during the COVID-19 pandemic have not been clarified. This study aims to explore the factors associated with bonding between fathers and infants during the COVID-19 pandemic.

## Results

### Participants characteristics

A total of 1055 men were included in the analysis and divided into two groups: primiparas and multiparas. Of the 1055 men, 521 (49.4%) had a primipara partner or wife, and 534 (50.6%) had a multipara partner or wife. Participants’ characteristics are presented in Table 1. The majority of the participants in the primipara group wanted pregnancies; further, among this group, more emergency cesarean sections were performed, the child was more likely to be admitted to the NICU, and the child was more likely to be a girl. Table 1 shows a comparison of the outcomes between the two study groups. No significant differences were found between the two groups in terms of a Lack of Affection, and Anger and Rejection.

**Table 1:** Basic characteristics of participants.

	Primipara group (N = 521)	Multipara group (N = 534)	p value
Age of men (father): mean (SD)	35.68 (5.23)	35.41 (5.33)	0.415
Academic history: n (%)			
Junior or high school	47 (9.0)	62 (11.6)	0.203
Junior college	53 (10.2)	68 (12.7)	
University or college	339 (65.1)	333 (62.4)	
Graduate school	82 (15.7)	71 (13.3)	
Mode of delivery: n (%)			
Vaginal delivery	416 (79.8)	429 (80.3)	< 0.001*
Emergency CS	71 (13.6)	25 (4.7)	
Elective CS	34 (6.5)	80 (15.0)	
Gestational week of delivery: mean (SD)	38.98 (2.23)	38.83 (1.98)	0.238
Preterm birth: n (%)			
Yes	47 (9.0)	52 (9.7)	0.752
No	474 (91.0)	482 (90.3)	
Low birthweight: n (%)			
Yes	42 (8.1)	33 (6.2)	0.281
No	479 (91.9)	501 (93.8)	
Birth weight (g): mean (SD)	2995.47 (423.01)	3056.34 (397.44)	0.016*
Youngest child’s admission history to NICU: n (%)			
Yes	61 (11.7)	38 (7.1)	0.014*
No	460 (88.3)	496 (92.9)	
Biological sex of youngest child: n (%)			
Girl	267 (51.2)	240 (44.9)	0.042*
Boy	254 (48.8)	294 (55.1)	
Age of youngest child (month): mean (SD)	9.13 (4.92)	8.93 (4.83)	0.523
Wanted pregnancy: n (%)			
Yes	501 (96.2)	496 (92.9)	0.028*
No	20 (3.8)	38 (7.1)	
EPDS: mean (SD)	6.63 (5.51)	6.77 (5.60)	0.678
Family APGAR: mean (SD)	7.18 (2.93)	7.01 (2.95)	0.361
MIBS_LA: mean (SD)	1.52 (2.20)	1.65 (2.25)	0.345
MIBS_AR: mean (SD)	2.17 (2.13)	2.16 (2.33)	0.954

*SD* standard deviation, *CS* cesarean section, *NICU* neonatal intensive care unit, *EPDS* Edinburgh Postnatal Depression Scale, *Family APGAR* family adaptability, partnership, growth, affection, and resolve, *MIBS* Mother-to-infant Bonding Scale, *LA* lack of affection, *A*R anger and rejection. A test for differences between primipara and multipara groups. *Significant at 0.05 level.

### Factors associated with father–infant bonding

Linear regression analysis was used to identify factors associated with father–infant bonding for the two groups during the COVID-19 pandemic. After adjusting for age (continuous) and education level (reference graduate school), variables shown to be associated with prior literature were added forward–backward stepwise. Table 2 presents the variables associated with Lack of Affection in the primipara group. Variables such as postpartum depression [B = 0.08; confidence interval (CI) 0.05–0.11], difficulty taking care of children at home because the partner or wife was at home working or on maternity leave (B = 0.53; CI 0.29–0.76) and the youngest child’s admission to the NICU (B = 0.75; CI 0.27–1.23) were associated with a lack of affection toward their children (Table 2). However, variables such as better family functioning (B = − 0.16; CI − 0.24 to − 0.08), partner or wife’s response to the father’s feelings (B = − 0.50; CI − 0.81 to − 0.20), insult by the partner or wife after January 2021 (B = -1.38; CI − 1.95 to − 0.81) and worry over accusations of being infected with COVID-19 (B = − 0.75; CI − 1.34 to − 0.17) tended to influence affection toward their children (Table 2).

**Table 2:** Factors associated with MIBS-J_LA in primipara group (n = 521).

	Unstandardized regression coefficients (B)	95% confidence intervals	
Age of men (father) [continuous]	− 0.02	− 0.05	0.01
Academic history			
Junior or high school [0,1]	− 0.18	− 0.82	0.46
Junior college [0,1]	− 0.06	− 0.67	0.56
University or college [0,1]	− 0.15	− 0.58	0.27
Graduate school (ref.)			
EPDS [continuous]*	0.08	0.05	0.11
Family APGAR [continuous]*	− 0.16	− 0.24	− 0.08
Childhood experience of parent swearing at them or insulting them [0,1]*	− 0.89	− 1.46	− 0.33
Childhood experience of parents separated or divorced [0,1]*	− 0.59	− 1.14	− 0.03
Wanted pregnancy [0,1]*	− 0.85	− 1.66	− 0.04
Partner or wife's responding to feelings of participants [1: Never–4: Often]*	− 0.50	− 0.81	− 0.20
Difficulty in taking care of children at home because partner (wife) is home working work or on maternity leave [1: Never–4: Always]*	0.53	0.29	0.76
Insulted by partner (wife) after January 2021 [0,1]*	− 1.38	− 1.95	− 0.81
Physical injury because of a fight with partner or wife after January 2021 [0,1]	1.59	− 0.02	3.20
Worried about accusations of being infected with COVID-19 [0,1]*	− 0.75	− 1.34	− 0.17
Age of youngest child (month) [continuous]*	− 0.03	− 0.06	0.00
Youngest child’s admission history to NICU l [0,1]*	0.75	0.27	1.23
Youngest child has been diagnosed with a language delay [0,1]*	1.74	0.26	3.21
Shutting their child out the house [1: Never–4: Often]*	0.64	0.06	1.23

*MIBS* Mother-to-infant Bonding Scale, *LA* lack of affection, *EPDS* Edinburgh Postnatal Depression Scale, *Family APGAR* family adaptability, partnership, growth, affection, and resolve, *NICU* neonatal intensive care unit. *Significant at 0.05 level.

Table 3 shows the variables associated with Lack of Affection in the multipara group. Variables such as postpartum depression (B = 0.05; CI 0.02–0.09), difficulty taking care of children at home because the partner or wife was home working or on maternity leave (B = 0.38; CI 0.14–0.62), and shutting their children out of the house (B = 0.68; CI 0.24–1.12) were associated with problems in lack of affection toward their children. Variables such as support from partner or wife (B = − 0.19; p = 0.002), better family functioning (B = − 0.14; CI − 0.23 to − 0.04) and wanted pregnancy (B = − 1.09; CI − 1.72 to − 0.46) tended to influence affection toward their children (Table 3).

**Table 3:** Factors associated with MIBS-J_LA in multipara group (n = 534).

	Unstandardized regression coefficients (B)	95% confidence intervals	
Age of men (father) [continuous]	− 0.03	− 0.06	0.00
Academic history			
Junior or high school	− 0.22	− 0.87	0.43
Junior college	− 0.26	− 0.90	0.37
University or college	− 0.03	− 0.52	0.46
Graduate school (ref.)			
EPDS [continuous]*	0.05	0.02	0.09
Family APGAR [continuous]*	− 0.14	− 0.23	− 0.04
Wanted pregnancy [0,1]*	− 1.09	− 1.72	− 0.46
Support from partner (wife) [1: Never–4: Often]*	− 0.57	− 0.94	− 0.21
Difficulty in taking care of children at home because partner (wife) is home on work or maternity leave [1: Never–4: Always]*	0.38	0.14	0.62
Fear of COVID-19 [1: Strongly Disagree–5: Strongly Agree]*	− 0.20	− 0.34	− 0.05
Forced to have sexual intercourse by partner (wife) without consent after January 2021 [0,1]	1.46	0.00	2.91
Emergency cesarean section [0,1]*	0.95	0.18	1.72
Shutting their children out the house [1: Never–4: Often]*	0.68	0.24	1.12

*MIBS* Mother-to–Infant Bonding Scale, *LA* lack of affection, *EPDS* Edinburgh Postnatal Depression Scale, *Family APGAR* Family Adaptability, Partnership, Growth, Affection, and Resolve. *Significant at 0.05 level.

The variables associated with Anger and Rejection in the primipara group are presented in Table 4. Variables such as postpartum depression (B = 0.13; CI 0.09–0.16), scolding their children loudly (B = 0.70; CI 0.39–1.01), feeling troubled or anxious about family relationships (B = 0.88; CI 0.47–1.29), difficulty taking care of children at home because the partner or wife was home working or on maternity leave (B = 0.46; CI 0.23–0.70) were associated with father–infant bonding dysfunction due to anger and rejection. Variables such as better family functioning (β = − 0.14; p = 0.001) and insult by the partner or wife after January 2021 (B = − 0.79; CI − 1.34 to − 0.24) made the fathers less likely to show anger and rejection toward children.

**Table 4:** Factors associated with MIBS-J_AR in primipara group (n = 521).

	Unstandardized regression coefficients (B)	95% confidence intervals	
Age of men (father) [continuous]	− 0.02	− 0.05	0.01
Academic history			
Junior or high school	0.21	− 0.43	0.84
Junior college	− 0.14	− 0.75	0.47
University or college	0.03	− 0.39	0.46
Graduate school (ref.)			
EPDS [continuous]*	0.13	0.09	0.16
Family APGAR [continuous]*	− 0.10	− 0.16	− 0.04
Childhood experience with financial hardship [0,1]*	− 0.56	− 1.08	− 0.05
Troubled or anxious about family relationships [0,1]*	0.88	0.47	1.29
Difficulty in taking care of children at home because partner (wife) is home working or on maternity leave [1: Never–4: Always]*	0.46	0.23	0.70
Insulted by partner (wife) after January 2021 [0,1]*	− 0.79	− 1.34	− 0.24
Physically assaulted by partner (wife) after January 2021 [0,1]*	1.81	0.54	3.08
Scolding their child loudly at home [1: Never–4: Often]*	0.70	0.39	1.01

*MIBS* Mother-to-Infant Bonding Scale, *AR* anger and rejection, *EPDS* Edinburgh Postnatal Depression Scale, *Family APGAR* family adaptability, partnership, growth, affection, and resolve. *Significant at 0.05 level.

Table 5 presents the variables associated with Anger and Rejection in the multiparous group. Although variables such as postpartum depression (B = 0.14; CI 0.10–0.17), not feeding their children (B = 1.05; CI 0.49–1.61) and ignoring their children (B = 0.82; CI 0.38–1.25) were associated with father–infant bonding dysfunction due to anger and rejection. Variables such as feeling uneasy about others’ behavior regarding infection prevention after January 2021 (B = − 0.49; CI − 0.86 to − 010), spending more time with the children after COVID-19 (B = − 0.19; CI − 0.35 to − 0.03) and having co-workers or neighbors infected with COVID-19 after January 2021 (B = − 0.48; CI − 0.88 to − 0.08) made the fathers less likely to show anger and rejection toward the children.

**Table 5:** Factors associated with MIBS-J_AR in multipara group (n = 534).

	Unstandardized regression coefficients (B)	95% confidence intervals	
Age of men (father) [continuous]	0.02	− 0.01	0.05
Academic history			
Junior or high school	− 0.09	− 0.75	0.56
Junior college*	0.64	0.00	1.28
University or college	0.27	− 0.22	0.76
Graduate school (ref.)			
EPDS [continuous]*	0.14	0.10	0.17
Family APGAR [continuous]*	− 0.06	− 0.12	− 0.00
Childhood sexual abuse [0,1]*	2.59	0.67	4.51
Troubled or anxious about pregnancy or childbirth [0,1]*	1.08	0.48	1.68
Co-workers or neighbors infected with COVID-19 after January 2021 [0,1]*	− 0.48	− 0.88	− 0.08
Spending more time with children after COVID-19 [1: Never–4: Always]*	− 0.19	− 0.35	− 0.03
Feeling uneasy about others' behavior regarding infection prevention after January 2021 [0,1]*	− 0.48	− 0.86	− 0.10
Not feeding their children at home [1: Never–4: Often]*	1.05	0.49	1.61
Ignoring their children at home [1: Never–4: Often]*	0.82	0.38	1.25

*MIBS* Mother-to–Infant Bonding Scale, *AR* anger and rejection, *EPDS* Edinburgh Postnatal Depression Scale, *Family APGAR* Family Adaptability, Partnership, Growth, Affection, and Resolve. *Significant at 0.05 level.

## Discussion

The search for factors affecting father–infant bonding during the COVID-19 pandemic revealed evidence from previous studies and relationships with factors related to COVID-19 infection. Regarding factors related to COVID-19, it was found that caring for the child while the partner or wife was at home working or on maternity leave had a negative impact on bonding between father and infant, while worry or fear related to infection with COVID-19 had no negative impact on bonding.

This study was conducted while COVID-19 was spreading across the country. Therefore, there may have been more occasions than usual for fathers to look after their children at home even when their wives or partners were also home. Men who encountered difficulties in caring for their children under these circumstances exhibited bonding difficulties in both the “Lack of Affection” and “Anger and Rejection” categories for the primipara group, and in the “Lack of Affection” category for the multipara group. This result may indicate a negative effect of the COVID-19 pandemic on father–infant bonding, as hypothesized at the beginning of this paper. During the COVID-19 pandemic, men were more prone to social isolation and loneliness^19^. It is likely that men, especially those who usually spend little time involved in child rearing, are more likely to feel anxious and stressed about taking care of their children under such circumstances. Postpartum anxiety and stress in fathers have been found to affect father–infant bonding^7,8,29^, and similar results were obtained in the present study. Regardless of the primipara or multipara group, there was an association between paternal postpartum depression and difficulties in the father–child relationship (Lack of Affection and Anger and Rejection). In the multipara group, fathers’ worries and anxieties about pregnancy and childbirth were associated with Anger and Rejection toward their children.

Previous literature has shown that the poor quality of these relationships with partners also results in poor bonding between parents and their children^9^. In the current study, variables representing partner relationships include, “Support from partner (wife),” “Partner or wife's responding to feelings of participants”, “Insulted by partner (wife) after January 2021”, “Physically assaulted by partner (wife) after January 2021”, and “Physical injury because of a fight with partner or wife after January 2021”. Regarding the relationship between these variables and father–infant bonding, in the primipara group, the more frequently the partner or wife responded to the father’s feelings, and in the multipara group, the more frequently the father received support from his partner, the more likely they were to show affection during father–infant bonding. In the primipara group, physical violence from the wife or partner was significantly associated with anger and rejection toward the children, and in the multipara group, sexual intercourse without the consent of the wife or partner was significantly associated with lack of affection toward the children. These results were consistent with those of previous studies. However, regarding the experience of being insulted by their partners, the primiparous group was more likely to show affection toward their children and less likely to show anger and rejection. To the best of our knowledge, no prior studies have explained this.

Regarding family functioning, Family APGAR was found to be a predictor of mother–infant bonding impairments in a study targeting mothers^30^. The present study also used the Family APGAR and found that in both groups, better family functioning tended to indicate more affection toward the child and less anger and rejection. Conversely, distress and anxiety in family relationships tended to indicate anger and rejection toward the children.

The study also found a positive relationship between the COVID-19 pandemic and father–infant bonding. Men in the multipara group who spent more time with their children due to the spread of COVID-19 infection tended to feel less anger and rejection.

Worry and fear related to infection with COVID-19 did not indicate any problems with father–infant bonding. In the primiparous group, worry about accusations of being infected with COVID-19 was associated with fathers’ affection toward their children. In the multipara group, greater fear of COVID-19 infection also influenced affection toward the children, and anxiety over one’s coworkers or neighbors being infected with COVID-19 or about the actions of others in preventing infection was less likely to be expressed as anger or rejection toward the children. Previous literature on COVID-19 found that living in an area with high rates of COVID-19 infection and mortality and high levels of paternal anxiety were associated with paternal parenting stress, indicating a discrepancy between the previous literature and the results of this study^15^. These anxieties and fears may be explained if they are related to the expressed desire to protect children from COVID-19.

As for fathers’ childhood experiences, in the primipara group, parents’ divorce and experiences with parents saying hurtful things or insulting them were associated with affection toward their children, and experiences of financial hardship tended not to influence anger and rejection. In the multipara group, experiences of sexual abuse during childhood were significantly associated with anger and rejection toward the children. To our knowledge, there is no evidence of any relationship between fathers’ adverse childhood experiences and bonding with their children; however, there is evidence that fathers’ adverse childhood experiences are associated with the perception of their child’s behavior after birth and the intergenerational transmission of child abuse. Although the relationship between fathers’ adverse childhood experiences and the perception of their child's behavior is mediated by partner disharmony and related to the perception of their child’s behavior in the postpartum period, fathers’ adverse childhood experiences do not directly lead to the postpartum perception of their child’s behavior^31^. Regarding parents’ experiences with childhood maltreatment, it is known that the children of mothers who have experienced childhood maltreatment are at an increased risk for socioemotional problems and maltreatment, coinciding with the mother’s antenatal depression^32^. As the relationship between fathers’ adverse childhood experiences and bonding with their children may be complicated due to the number and nature of fathers’ adverse childhood experiences and their relationships with their partners, more detailed research is needed.

Regarding the factors related to child maltreatment, shutting children out of the house was significantly associated with a lack of affection in both groups. In the primipara group, the variable of scolding their children loudly at home was significantly associated with anger and rejection toward the children, while in the multipara group, the variables of not feeding their children and ignoring the children at home were significantly associated with anger and rejection toward the children. According to the World Health Organization^33^, parental characteristics that may increase the risk of child maltreatment include difficulty in bonding with their newborns. These results were consistent those of previous studies.

This study had several limitations. We recruited participants through an Internet research organization, meaning that our sample was limited to Internet users. Therefore, the sample may not represent the entire population of men whose partners have given birth. The present study used the MIBS as a measure of father–infant bonding. Although this scale has been validated for its factor structure of the scale in fathers, its construct validity has not been assessed, which is another limitation.

In conclusion, this study revealed the factors influencing father–infant bonding during the COVID-19 pandemic. The COVID-19 pandemic has increased the amount of time both fathers and mothers spent at home; thus, their relationships and family functioning may have affected father–infant bonding. The study also found that worry and fear related to COVID-19 infection did not negatively affect the bonding between fathers and their children. However, further detailed research that considers the infection status in residential areas is required.

## Methods

### Study design

This cross-sectional study used data from the Japan COVID-19 and Society Internet Survey (JACSIS), a nationwide cross-sectional survey conducted in 2021^34^. Additionally, we used data from pregnancy and maternity surveys, which include data on fathers whose partners were currently pregnant or postpartum^13^. This study was reported in accordance with the Strengthening the Reporting of Observational Studies in Epidemiology (STROBE) checklist^35^ (Supplementary Information).

### Sample

**Figure 1:**
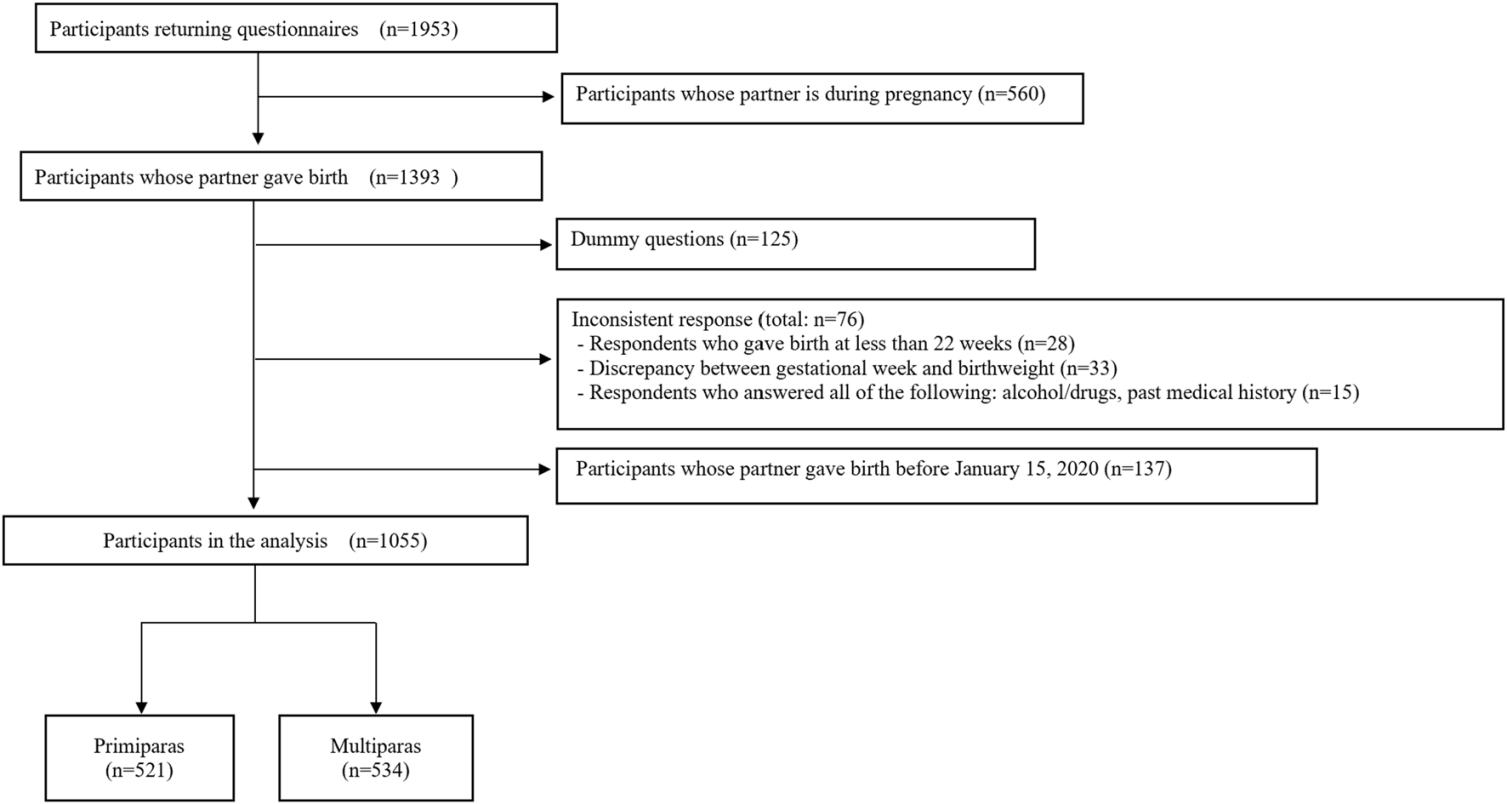
Flow chart.

The study samples for this survey were retrieved from the pooled panels of the Internet research company Rakuten Insight, Inc., Hong Kong, which holds approximately 2.3 million panelists who voluntarily registered in exchange for small incentive points when completing questionnaires^13,34^. Figure 1 presents a flowchart of the data extraction process. We excluded those whose youngest child had not yet been born (*n* = 560). The following were also considered invalid responses and excluded from the sample: respondents who did not select a specific option when asked to do so in a dummy question (*n* = 125); respondents who reported less than 22 weeks of delivery (*n* = 28); respondents who reported data on the apparent imbalance between weeks of delivery and birth weight (*n* = 33); respondents who reported abusing all seven substances (alcohol, sleeping medications, opioids, sniffing paint thinner, legal-high drugs, marijuana, and cocaine/heroin); and respondents who selected all 16 medical histories on the list (*n* = 15). To identify father–infant bonding during the COVID-19 pandemic, we included data after the first COVID-19 cases were reported in Japan, which were the partners of those who gave birth between January 16, 2020, and August 18, 2021 (*n* = 1055). No missing data were observed.

### Variable measurement

#### Outcome

The Mother-to-Infant Bonding Scale (MIBS) was used to measure father-to-infant bonding. The Japanese version of Mother-to-Infant Bonding Scale (MIBS-J) was developed by Yoshida et al. (2012) based on the Mother-infant Bonding Questionnaire by Kumar^36^. This scale was originally developed to measure bonding between mothers and children but has also been validated for fathers^10^. The scale consists of 10 items, such as “I feel loving towards my baby”, which are answered on a four-point scale of “almost always strongly agree”, “sometimes strongly agree”, “sometimes slightly agree” and “never agree^10^”^8^. The maximum score was 30 points, with higher scores corresponding to weaker bonding^10^.

Kitamura et al.^37^ reported that the MIBS-J was structured into two factors, “Anger and Rejection” (items 2, 3, 5 and 7) and “Lack of Affection” (items 1, 6, 8, and 10) by exploratory factor analysis. As these are different aspects of the fathers’ bonding^23,37^, MIBS-J scores in this study were calculated separately for Anger and Rejection and Lack of Affection in this study.

#### Covariates

Previous studies have found that the father’s presence at birth^11,12^, participation in paternity classes^11–14^, number of children^8^, parental leave status^13^, and developmental delay in the child^38^ affected father–infant bonding. As paternal attributes, medical history including depression^29^ and the Edinburgh Postnatal Depression Scale^10,29,39^ were reported as factors associated with father–infant bonding. The Edinburgh Postnatal Depression Scale is a 10-item self-report scale for screening postpartum depression^39^ in mothers and fathers, with a cut-off score of 8/9 and has been found to identify probable postnatal depression^40^. Partner relationships^8,9,14^ and partner violence^29^, anxiety^7^, and fathers’ adverse childhood experiences^20,41,42^ have also been shown to be associated with father–infant bonding.

#### Predictors

We describe the predictors conceptually separately from the covariates to convey the purpose of this study more clearly. The following were used as predictors: desired pregnancy, child abuse-related behavior, and variables such as emergency cesarean section, premature birth, low birth weight and Neonatal Intensive Care Unit (NICU) admission, which were considered to potentially impede early contact between the infant and father. Family adaptability, partnership, growth, affection, and resolve (Family APGAR) also included as predictors. Family APGAR is a five-item scale used to determine self-reported family dysfunction, with higher scores indicating highly functional families^43^. The variables were related to the recent COVID-19 pandemic, history of COVID-19 infection, anxiety about COVID-19 infection, and the impact of the COVID-19 pandemic on childcare and work, including teleworking.

### Statistical analysis

The study participants were divided into two groups, depending on their partners’ parity because these differences have been reported as effect modifiers of father–infant bonding^8^. Descriptive statistics for all variables included in this study were mean standard deviation (SD) and sample size (n, %). For comparisons between the two groups, the student’s t-test was used for continuous variables and a chi-square test was used for categorical variables. The predictors and covariates were entered into the linear (Gauss–Markov-type) regression model as follows. In the first step, age and education level were entered using the forced entry method to examine associations with factors from the MIBS-J as demographic variables. Then, in Step 2, the above variables, which have been shown to be associated with father–infant bonding in the previous studies, were added to the Step 1 model with forward–backward stepwise variable selection approach while keeping the variables in the Step 1. The input/removal criteria during the stepwise iterations were measured based on the distribution of F-statistics with 5% for the input and 10% for the removal, respectively. Two-sided p value of < 0.05 was considered statistically significant. Multicollinearity was tested using variance inflation factor (VIF), and no multicollinearity was observed. Statistical analysis was conducted using IBM SPSS statistics Version 23.

### Ethical approval

This study was approved by the Bioethics Review Committee of Osaka International Cancer Institute, Japan (approval number: 20084). Informed consent was obtained electronically from all participants prior to the survey. Data were collected anonymously. This study was performed in accordance with the ethical guidelines for medical and health research involving human subjects enforced by the Japanese Ministry of Health, Labour, and Welfare, and the Declaration of Helsinki.

### Supplementary Information

Supplementary Information.

## Data Availability

The datasets generated during and/or analyzed during the current study are available from the corresponding author on reasonable request.
